# Management of Secondary Poor Response to Botulinum Toxin in Cervical Dystonia: A Multicenter Audit

**DOI:** 10.1002/mdc3.13181

**Published:** 2021-03-16

**Authors:** Harry Tucker, Foster Osei‐Poku, Diane Ashton, Rachael Lally, Aaron Jesuthasan, Anna Latorre, Kailash P. Bhatia, Jane E. Alty, Christopher Kobylecki

**Affiliations:** ^1^ Department of Neurology, Manchester Centre for Clinical Neurosciences Salford Royal NHS Foundation Trust Salford United Kingdom; ^2^ Leeds Centre for Neurosciences Leeds Teaching Hospitals NHS Trust Leeds United Kingdom; ^3^ Newcastle University Newcastle upon Tyne United Kingdom; ^4^ Institute of Neurology UCL London United Kingdom; ^5^ Wicking Dementia Research and Education Centre, College of Health and Medicine University of Tasmania Hobart Tasmania Australia; ^6^ Manchester Academic Health Sciences Centre University of Manchester Manchester United Kingdom

**Keywords:** cervical dystonia, botulinum toxin, secondary non‐response

## Abstract

**Background:**

Botulinum toxin A (BoNT‐A) is an effective treatment for cervical dystonia. Nevertheless, up to 30% to 40% patients discontinue treatment, often because of poor response. The British Neurotoxin Network (BNN) recently published guidelines on the management of poor response to BoNT‐A in cervical dystonia, but adherence to these guidelines has not yet been assessed.

**Objectives:**

To assess adherence to and usefulness of BNN guidelines in clinical practice.

**Methods:**

We undertook a retrospective medical notes audit of adherence to the BNN guidelines in 3 United Kingdom tertiary neurosciences centers.

**Results:**

Of 76 patients identified with poor response, 42 (55%) had a suboptimal response and, following BNN recommendations, 25 of them (60%) responded to adjustments in BoNT dose, muscle selection or injection technique. Of the remaining 34 (45%) patients with no BoNT response, 20 (59%) were tested for immune resistance, 8 (40%) of whom showed resistance. Fourteen (18%) of all patients were switched to BoNT‐B, and 27 (36%) were referred for deep brain stimulation surgery. In those not immune to BoNT‐A, clinical improvement was seen in 5 (41%) after adjusting their dose and injection technique.

**Conclusion:**

Our audit shows that optimizing BoNT dose or injection strategy largely led to improvements in those with suboptimal response and in those reporting no response without resistance. It would be helpful to standardize investigations of potential resistance in those with no therapeutic response.

Cervical dystonia (CD) is a movement disorder characterized by involuntary muscle contractions causing abnormal postures of the head and neck.^1^ Injection with botulinum toxin type A (BoNT‐A) is an effective first‐line treatment for dystonic movements, with level A evidence.^1^ However, up to 30% to 40% of patients with CD discontinue long‐term treatment, often because of perceived lack of response.^2^ Poor response can be classified as primary, where BoNT‐A injections have never helped or, more commonly, as secondary defined as failure to respond following previous successful treatment.^3^ Secondary non‐response to treatment has been identified as a cause of BoNT discontinuation in 13.6% (range 3.9%–38%) of patients across multiple studies.^2^ There are a number of potential causes for this, with suboptimal BoNT dose or muscle selection representing the most common causes in 1 series.^4^ Immune resistance to BoNT‐A is another recognized cause, although the exact prevalence of this phenomenon is hard to quantify.^5^

Marion and colleagues published consensus guidance from the British Neurotoxin Network (BNN) in 2016 with the aim of improving the management of patients with CD showing a poor response to BoNT‐A injections.^3^ These guidelines are partly based on a survey of practice in experienced neurologists treating patients with CD.^6^ The guidance recommends clinicians first distinguish between a suboptimal response and no response to BoNT‐A. In those with suboptimal response, the BNN guidelines recommend clinicians consider revision of dose, muscle selection, and use of electromyography (EMG) guided injections. In those with no therapeutic response, assessment for resistance to BoNT‐A is recommended alongside the measures described above.^3^ Where resistance is identified, switching to BoNT‐B or a treatment break is suggested. Referral for deep brain stimulation (DBS) surgery for patients with ongoing refractory CD is also proposed.

We sought to establish adherence to the BNN guidelines in 3 large dystonia services based at United Kingdom (UK) tertiary neuroscience centers.

## Methods

We carried out a retrospective audit of clinic notes from dystonia clinics at Manchester Centre for Clinical Neurosciences, Salford, Leeds Centre for Neurosciences, Leeds, and University College London Hospitals, London. This was approved by the respective audit committees and formal ethical approval was not required. Data from patients with CD who had been identified as having secondary non‐response to BoNT‐A injections were collected using a standardized proforma encompassing the steps outlined in the BNN guidelines. We defined poor response as per the BNN guidelines as “two consecutive treatments with suboptimal response, where the patient has previously received a minimum of two successful injection cycles”.^3^ Because standardized clinical scores to define response were not available in all cases, we defined poor response according to patient report. The proportion of patients experiencing suboptimal clinical response, defined as a partial but unsatisfactory effect, or no response to BoNT‐A was first determined, before we evaluated the proportion that subsequently showed a response to measures outlined by the guidance.

## Results

We evaluated notes from 76 patients with CD who had been identified as having a poor response to BoNT between 2012 to 2017. The percentage of the whole clinic population of CD patients was as follows: Salford 45/700 (6.4%), Leeds 25/196 (12.7%), London 6/360 (1.7%). The mean age was 60 ± 12 years (range 32–86), 45 (59%) were female and the median duration since dystonia symptom onset was 12 years (interquartile range [IQR] 7–17). All patients apart from 1 had idiopathic isolated cervical dystonia; 1 had acquired dystonia. Dystonia in other body regions was seen in 3 patients (limb tremor 1, limb tremor + blepharospasm 1, oromandibular 1). The predominant type of dystonic movement was torticollis in 39 (51%), laterocollis in 6 (8%), retrocollis in 2 (3%), mixed in 16 (21%). In 11 cases, this information was not available. Dystonic tremor was documented in 27 cases (36%). Patient assessment was done by movement disorder specialist neurologists or dystonia specialist nurses in all cases, although the same practitioner did not always assess the same patient through their whole treatment course. There were no differences between centers in terms of experience with BoNT treatment or dosing.

A poor BoNT response occurred after a median latency of 6 years from treatment initiation (IQR 2–12). The majority (52; 68%) were receiving treatment with abobotulinumtoxinA (Dysport) at the time of non‐response (12 with onabotulinumtoxinA [BOTOX], 7 with incobotulinumtoxinA [Xeomin], and 4 with rimabotulinumtoxinB [Neurobloc]). In 1 case, the BoNT preparation was not documented.

**FIG. 1:**
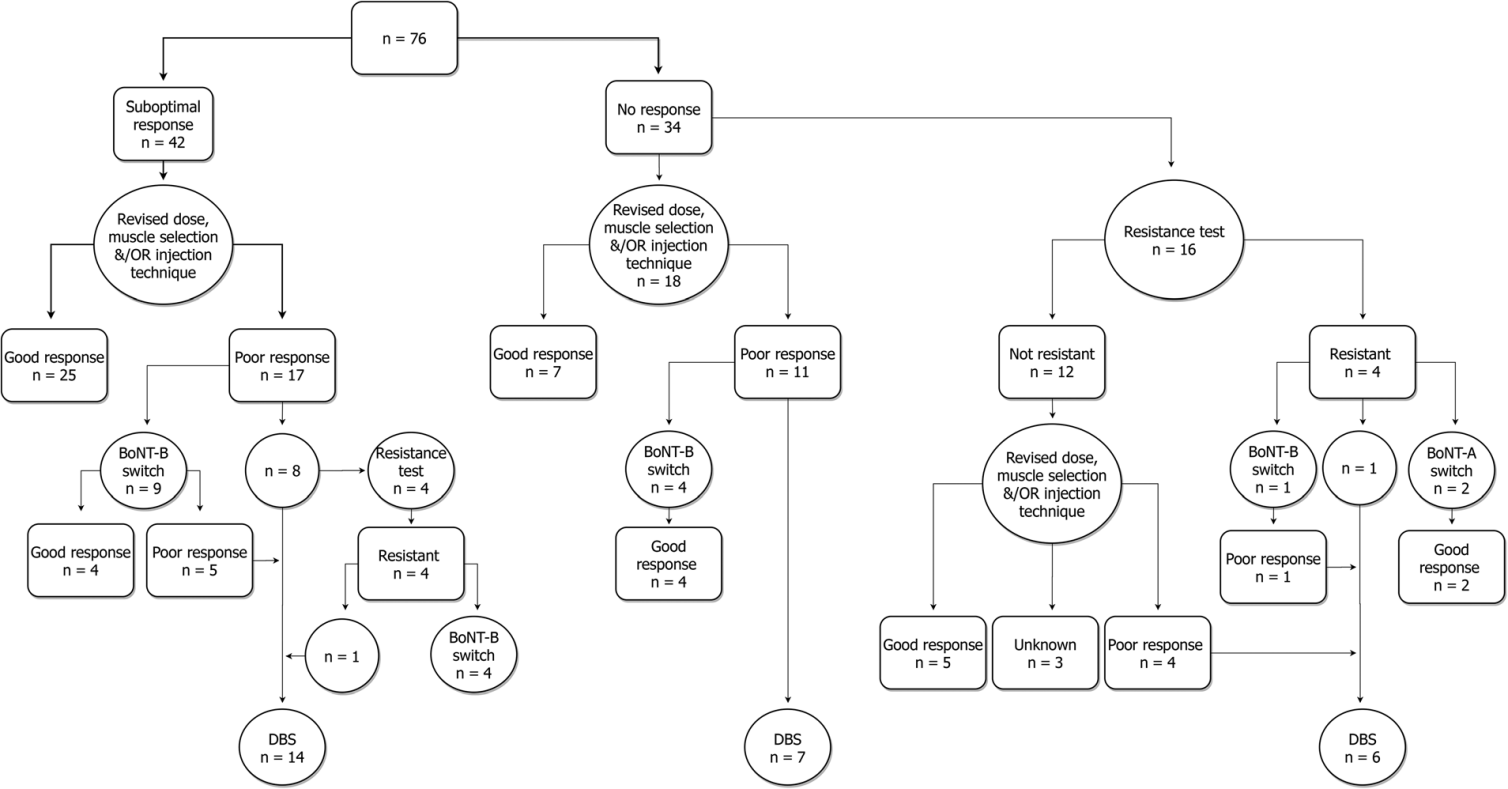
Flow chart indicating adherence to the British neurotoxin network guidelines.^3^ BoNT, botulinum toxin; DBS, deep brain stimulation surgery.

The assessment of patients according to the BNN guidelines is shown in Figure 1. A suboptimal response to BoNT, defined as a partial but unsatisfactory effect, was seen in 42 patients (55%). Twenty‐five of these (60%) exhibited an improvement in response following revisions to BoNT dose, muscle selection and/or injection technique. EMG was used to guide injections in 52 cases (68%). In patients who continued to have poor response despite changes recommended by BNN guidance, 4 cases underwent formal assessment for immune resistance, whereas 9 patients were switched directly to BoNT‐B. A total of 14 were referred for DBS either directly or having first tried BoNT‐B.

In 34 patients assessed as obtaining no therapeutic effect from BoNT‐A, the recommendation to perform a test of immune resistance was followed in 20 cases (59%). The muscle used for this was as follows: frontalis in 11 patients, extensor digitorum brevis (EDB) in 1 (the details of these are described in the BNN guidelines) ^3^ and abductor digiti minimi (ADM) in 8 patients. This latter test involves injection of 20 units BOTOX/Xeomin or 100 units Dysport into the ADM, with assessment 10 to 14 days later for weakness. Functional resistance to BoNT‐A was identified in 40% of those tested, the majority of whom were switched to BoNT‐B. In those not resistant to BoNT‐A, clinical improvement was seen in 41% following revisions of dose or muscle selection. The majority (61%) of remaining patients in whom testing for immune resistance was not carried out showed a poor response to revisions of dose or technique. Most were empirically switched to BoNT‐B or referred for DBS. In total, 14 of our cohort (18%) were switched to BoNT‐B and 27 (36%) were referred for DBS. At the time of writing, DBS was performed in 7 cases.

In line with BNN guidance, additional oral therapy for dystonia was prescribed in 53 cases (70%), and 36 (47%) were referred for physiotherapy. There was evidence of additional pain management interventions in 37 (49%).

## Discussion

This is the first published audit of clinical practice surrounding the management of patients with CD and poor BoNT response against recently published guidance from the BNN.^3^ The distribution of different subtypes of CD and dystonic tremor is in line with previous large cohort studies.^7^ In the majority of cases, the guidance on managing patients with suboptimal BoNT response was followed, 60% of whom achieved improvement with changes to BoNT dosing or muscle selection, including the use of EMG. This is in line with previous work indicating that incorrect muscle selection and BoNT dosage are the most common causes of BoNT failure.^4^ There is evidence that EMG‐guided injection of BoNT improves outcomes in patients with an unsatisfactory response,^8^, ^9^ but this technique was not used in all of our patients (despite EMG being available in all centers). Access to specialist EMG‐guided injection clinics could be a limiting step to further improve treatment outcomes.

The main deviation from the BNN guidelines is that 40% of patients with no response to BoNT did not have testing to determine potential immune resistance. Of those who did undergo such assessment, 8 of the 20 cases were found to be resistant to BoNT. Differences in approach to determining resistance were also observed between centers. The majority of patients underwent frontalis or EDB testing as recommended, although ADM testing was also carried out. This variation in approach likely reflects differences in training between centers.

The frequency of immune resistance as a source of secondary non‐response is variable in published series and depends on the methods used to ascertain resistance and whether these are done consistently.^4^, ^8^ Neutralizing antibodies (NABs) have been reported in 2.5% of a large series of CD patients treated with BoNT‐A, but were present in 9 of 17 cases with secondary non‐response.^10^ More recently, NABs to BoNT‐A were estimated to occur in 15% of CD patients after 5.6 years of BoNT‐A treatment,^5^ although these are not routinely tested in clinical practice, and their functional implications are not fully understood. Because testing for NABs is not routinely available, BNN guidance recommends clinical/functional methods of assessing immune resistance. It is of interest that 2 patients with resistance were switched to a different BoNT‐A preparation. Although there is some evidence for lower immunogenicity with incobotulinumtoxinA, data on long‐term outcomes following switch are not yet available. Our findings indicate the importance of a structured approach to determine functional resistance to BoNT‐A, which may help better identify the treatment pathway according to BNN guidance. In particular, a significant proportion of those not resistant to BoNT‐A derived good benefit from dose and injection adjustments, indicating that these changes can be beneficial even in those thought to be unresponsive. The long‐term effects of BoNT‐B need to be observed in patients with resistance, given its relatively high immunogenicity and potential for further resistance.^11^

Consistent with BNN guidance and findings from a large survey of medication use for dystonia,^12^ additional oral medications were prescribed in the majority of our patient cohort. Despite the limited evidence base for many of these in CD, this pattern reflects the complexity of managing CD patients with secondary non‐response. In addition, pain management and physiotherapy, recommended by the BNN guidance, were applied in a lower proportion of patients. Although pain is not a criterion for poor response in the BNN guidance per se, it may reflect increasing levels of complexity and comorbidities contributing to poor response. Variability in use of these services may reflect differences in access to therapies or patient/clinician preference. Despite the problems with establishing a clear evidence base for interventions such as physiotherapy for CD, it is used at some point by the majority of dystonia specialists.^6^

Our work has several limitations. First, because of its retrospective nature, details of response to BoNT treatments and other interventions were not standardized. The proportion of patients with poor response between centers was variable, but this is likely to reflect differences in methods of ascertainment and case‐mix. Further prospective studies could help clarify the proportions of patients in different services with poor response. Patient report was used to define poor response, and assessment at the peak of BoNT response, recommended in BNN guidance, was not always possible because of clinical pressures reflective of real‐life practice. We acknowledge that other definitions of secondary non‐response have been published that differ from the BNN definition, and require 3 or more cycles of poor response to BoNT treatment.^6^ Second, differences in practice between centers are seen. Significant variations with regard to the use of EMG‐guided injections and the identification of BoNT resistance are consistent with surveys from dystonia specialists.^6^ Our work did not address the role of ultrasound, although this is an expanding area of interest in CD and may help in optimizing BoNT treatment.^3^, ^13^ Finally, the eventual outcome of interventions such as DBS was not available in all cases. There is increasing evidence for the efficacy of globus pallidus pars interna (GPi) DBS for refractory CD,^14^ although we do not know the optimum number of cycles of treatment following the BNN modifications before DBS should be offered. Additionally, the relatively large number of patients referred for DBS reflects a good level of uptake amongst dystonia specialists and increasing acceptability to people with CD.

Our overall findings indicate that the proposed management of poor response to BoNT‐A outlined in the BNN guidelines is a useful framework for patient management. Clinicians should pay particular attention to optimizing muscle selection, dose, and injection technique, as these may help a significant number of patients. Access to EMG‐guidance and DBS services, as well as experience with different BoNT preparations are required for services to be able to better adhere to the published guidance. Furthermore, these will have significant implications for service development.

## Author Roles

(1) Research project: A. Conception, B. Organization, C. Execution; (2) Statistical Analysis: A. Design, B. Execution, C. Review and Critique; (3) Manuscript: A. Writing of the First Draft, B. Review and Critique.

H.T.: 1B, 1C, 2B, 3B

F.O‐P.: 1C, 3B

D.A.: 1B, 1C, 3B

R.L.: 1B, 1C, 3B

A.J.: 1B, 1C, 3B

A.L.: 1B, 3B

K.P.B.: 1C, 3B.

J.E.A.: 1A, 1B, 2C, 3B

C.K.: 1A, 1B, 1C, 2B, 2C, 3A, 3B

## Disclosures

### Ethical Compliance Statement

The authors confirm that the approval of an institutional review board was not required for this work. We confirm that we have read the Journal's position on issues involved in ethical publication and affirm that this work is consistent with those guidelines. The authors confirm that patient consent was not required for this work.

### Funding sources and conflict of interest

The authors report no sources of funding and no conflicts of interest.

### Financial disclosure for the previous 12 months

H.T. reports no disclosures. F. O‐P. reports no disclosures. D.A. reports no disclosures. R.L. reports no disclosures. A.J. reports no disclosures. A.L. reports no disclosures. K.P.B. reports grant support from Horizon 2020 EU grant 634,821 and honoraria/financial support to speak/attend meetings from GSK, Boehringer‐Ingelheim, Ipsen, Merz, Sun Pharma, Allergan, Teva, Lundbeck, and Orion pharmaceutical companies; royalties from Oxford University press and a stipend for MDCP editorship. J.E.A. reports grant support from NHMRC and honoraria/financial support to speak/attend meetings from UCB, Ipsen, Medtronic, Abbvie, Bial, Merz, and Allergan pharmaceutical/medical device companies; royalties from Taylor and Francis press; and stock ownership in Clearsky Medical Diagnostics. C.K. has received grants from Parkinson's UK and The Michael J. Fox Foundation; speaker fees from Britannia and Bial Pharma; support to attend international meetings from Abbvie, and advisory boards from Abbvie.

## References

[1] Albanese A , Abbruzzese G , Dressler D , et al. Practical guidance for CD management involving treatment of botulinum toxin: a consensus statement. J Neurol 2015;262:2201–2213. 25877834 10.1007/s00415-015-7703-x PMC4608989

[2] Jinnah HA , Comella CL , Perlmutter J , Lungu C , Hallett M , Dystonia Coalition Investigators . Longitudinal studies of botulinum toxin in cervical dystonia: why do patients discontinue therapy? Toxicon 2018;147:89–95. 28888929 10.1016/j.toxicon.2017.09.004 PMC5839920

[3] Marion MH , Humberstone M , Grunewald R , Wimalaratna S . British neurotoxin network recommendations for managing cervical dystonia in patients with a poor response to botulinum toxin. Pract Neurol 2016;16:288–295. 26976927 10.1136/practneurol-2015-001335 PMC4975836

[4] Jinnah HA , Goodmann E , Rosen AR , Evatt M , Freeman A , Factor S . Botulinum toxin treatment failures in cervical dystonia: causes, management, and outcomes. J Neurol 2016;263:1188–1194. 27113604 10.1007/s00415-016-8136-x PMC4904718

[5] Albrecht P , Jansen A , Lee JI , et al. High prevalence of neutralizing antibodies after long‐term botulinum neurotoxin therapy. Neurology 2019;92:e48–e54. 30464031 10.1212/WNL.0000000000006688

[6] Ferreira JJ , Bhidayasiri R , Colosimo C , Marti MJ , Zakine B , Maisonobe P . Survey of practices employed by neurologists for the definition and management of secondary non‐response to botulinum toxin in cervical dystonia. Funct Neurol 2012;27:225–230. 23597436 PMC3861346

[7] Misra VP , Colosimo C , Charles D , et al. INTEREST IN CD2, a global patient‐centred study of long‐term cervical dystonia treatment with botulinum toxin. J Neurol 2018;265:402–409. 29270685 10.1007/s00415-017-8698-2 PMC5808090

[8] Cordivari C , Misra VP , Vincent A , Catania S , Bhatia KP , Lees AJ . Secondary nonresponsiveness to botulinum toxin A in cervical dystonia: the role of electromyogram‐guided injections, botulinum toxin A antibody assay, and the extensor digitorum brevis test. Mov Disord 2006;21:1737–1741. 16874756 10.1002/mds.21051

[9] Nijmeijer SW , Koelman JH , Standaar TS , Postma M , Tijssen MA . Cervical dystonia: improved treatment response to botulinum toxin after referral to a tertiary Centre and the use of polymyography. Parkinsonism Relat Disord 2013;19:533–538. 23466060 10.1016/j.parkreldis.2013.01.018

[10] Kessler KR , Skutta M , Benecke R . Long‐term treatment of cervical dystonia with botulinum toxin A: efficacy, safety, and antibody frequency. German Dystonia study group. J Neurol 1999;246:265–274. 10367694 10.1007/s004150050345

[11] Berman B , Seeberger L , Kumar R . Long‐term safety, efficacy, dosing, and development of resistance with botulinum toxin type B in cervical dystonia. Mov Disord 2005;20:233–237. 15455449 10.1002/mds.20290

[12] Pirio Richardson S , Wegele AR , Skipper B , Deligtisch A , Jinnah HA , Dystonia Coalition I . Dystonia treatment: Patterns of medication use in an international cohort. Neurology 2017;88:543–550. 28077492 10.1212/WNL.0000000000003596 PMC5304465

[13] Castagna A , Albanese A . Management of cervical dystonia with botulinum neurotoxins and EMG/ultrasound guidance. Neurol Clin Pract 2019;9:64–73. 30859009 10.1212/CPJ.0000000000000568 PMC6382379

[14] Volkmann J , Mueller J , Deuschl G , et al. Pallidal neurostimulation in patients with medication‐refractory cervical dystonia: a randomised, sham‐controlled trial. Lancet Neurol 2014;13:875–884. 25127231 10.1016/S1474-4422(14)70143-7

